# Cross-stacked carbon nanotubes assisted self-separation of free-standing GaN substrates by hydride vapor phase epitaxy

**DOI:** 10.1038/srep28620

**Published:** 2016-06-24

**Authors:** Tongbo Wei, Jiankun Yang, Yang Wei, Ziqiang Huo, Xiaoli Ji, Yun Zhang, Junxi Wang, Jinmin Li, Shoushan Fan

**Affiliations:** 1 State Key Laboratory of Solid-State Lighting, Institute of Semiconductors, Chinese Academy of Sciences, Beijing, 100083, China; 2 State Key laboratory of Crystal Material, Shandong University, Jinan, 250100, China; 3 Department of Physics and Tsinghua–Foxconn Nanotechnology Research Center, Tsinghua University, Beijing 100084, China

## Abstract

We report a novel method to fabricate high quality 2-inch freestanding GaN substrate grown on cross-stacked carbon nanotubes (CSCNTs) coated sapphire by hydride vapor phase epitaxy (HVPE). As nanoscale masks, these CSCNTs can help weaken the interface connection and release the compressive stress by forming voids during fast coalescence and also block the propagation of threading dislocations (TDs). During the cool-down process, thermal stress-induced cracks are initiated at the CSCNTs interface with the help of air voids and propagated all over the films which leads to full self-separation of FS-GaN substrate. Raman and photoluminescence spectra further reveal the stress relief and crystalline improvement of GaN with CSCNTs. It is expected that the efficient, low cost and mass-producible technique may enable new applications for CNTs in nitride optoelectronic fields.

GaN materials have recently attracted considerable interests for their potential in diverse fields of optoelectronics and electronics materials and devices^1^,^2^,^3^. Due to a lack of commercially available GaN substrates with large diameters, most GaN-based devices, such as light emitting diodes (LEDs) and high electron mobility transistors (HEMTs), have been grown and fabricated on sapphire, SiC and Si substrates. However, hetero-epitaxial growth leads to strained epilayers with high defect densities and cracking problems caused by large lattice mismatch and thermal expansion coefficient differences between the epilayer and these substrates^4^,^5^. Therefore, the development of GaN substrates is imperative for addressing these problems in GaN-based applications. While growth of bulk GaN crystals by the ammono-thermal method has been progressing rapidly^6^,^7^, hydride vapor phase epitaxy (HVPE) is still considered the most promising tool due to its high growth rate and large size^8^,^9^,^10^. However, in the case of GaN bulk growth using HVPE, the growth is typically started on sapphire substrates. Removal of the sapphire from a thick GaN film remains a challenge because of sapphire’s large hardness and negligible etching rates wiry any etchant.

In order to reduce strain and bowing in the final quasi-substrate, and to facilitate the removal of the sapphire, different techniques have been explored. At the early stage, mechanical polishing of substrate^11^, laser lift-off and chemical etching lift-off^13^,^14^ technologies are widely used to produce free-standing (FS) GaN substrates. Nevertheless, these methods are time consuming and GaN layers are often cracked during polishing or lift-off. Subsequently, the self-separation technique of sapphire has been developed to eliminate cracking in both the polishing and the lift-off technique. Oshima *et al*. have suggested the void-assisted separation for the fabrication of FS-GaN, involving the formation of a TiN nanonet on the GaN template, but the *in situ* etching of GaN suffered from the catalytic effect of TiN is difficult to manipulate^15^. Lee *et al*. have presented an evaporable NH_4_Cl buffer layer to form voids during the high temperature stage to assist the self-separation of the thick film after cooling-down^16^. However, the introduction of the amorphous NH_4_Cl layer and the subsequent low temperature GaN cap layer would affect the crystalline quality of thick GaN films. More recently, Kobayashi *et al*. proposed a new micromechanical separation technique by inserting a hexagonal BN (h-BN) release layer between the GaN epilayer and the substrate^17^, but the method also introduced interfacial defects and hardly obtained larger sized GaN substrates. Therefore, it is important to develop simple new ways to grow and separate the GaN layer.

Because of the outstanding properties, such as low cost, high electrical and thermal conductivity, great mechanical strength and chemical inertness, carbon nanotubes (CNTs) have recently attracted considerable interest for various applications. In particular, superaligned CNTs films have already opened up the possibility of fabricating CNTs devices with uniform properties with the advantages of being low cost, mass-producible, scalable, etc.^18^,^19^. Without the transfer step, the CNTs can be readily assembled on target substrates by a simple coating process. Recently, we proposed CNTs patterned sapphire substrates for the improvement of quantum efficiency of LED devices^20^,^21^ by metal organic chemical vapor deposition (MOCVD). In this work, we report a new self-separation technique using aligned and cross-stacked CNTs (CSCNTs) as a nanoscale mask for the fabrication of FS-GaN substrate by HVPE. Selective growth on CSCNTs coated sapphire leads to the formation of many voids, which weaken the interface connection and thus cause self-separation of thick GaN films. Furthermore, the mechanism of voids formation and separation, as well as the improvement in crystalline quality of GaN films, are presented in detail.

## Experimental

**Figure 1:**
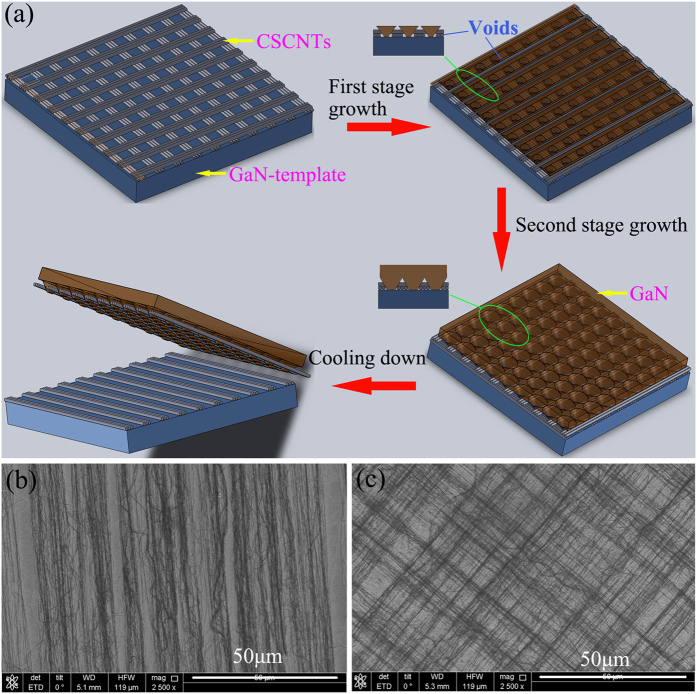
(**a**) Schematic illustrations of fabrication sequence of the self-separated FS-GaN substrate using CSCNTs. Surface morphology of CNTs patterned GaN template for (**b**) single layer CNTs and (**c**) double layer CSCNTs.

The fabrication sequences of self-separated FS-GaN films using CNTs as the interlayer are shown in Fig. 1(a). First, superaligned multi-walled carbon nanotube (MWCNT) arrays were grown on 4 inch silicon wafers by low pressure chemical vapor deposition (LPCVD) using a 5 nm iron film as a catalyst and acetylene as a precursor, as shown in our previous reports^18^,^19^,^22^. The diameter of the CNTs was about 10 nm. These special CNTs arrays are distinguished from the others for the improved CNT alighment and the strong inter-tube van der Waals interactions. Aligned CNTs films were continuously spun from the CNTs array due to the strong CNTs interactions with CNTs being aligned to the drawn direction. Such CNTs films were then coated on 3 μm-thick GaN template grown by MOCVD. To ensure that the CNTs were well-coated on the GaN template, the wafers were dipped into electronic grade ethanol solution and dried in air at room temperature. After the ethanol treatment, neighboring CNTs shrunk into bundles with a micrometer-scaled interval. The width of each bundle was around 1–3 μm and the height was about 100 nm, whereas the CNTs were tightly bonded together with 100–500 nm gaps in each bundle. Here, the CNTs with an alignment parallel to [11–20] of sapphire are named as single layer CNTs, as shown in Fig. 1(b). The CSCNTs are named as double layer CSCNTs shown in Fig. 1(c), in which the underlayer and upperlayer are parallel to [11–20] and [1–100] directions, respectively. Here, the height of the double layer CNTs is about 300 nm and CNTs are uniform on 2 inch template surface. After CNTs decorating on the GaN template, thick films were grown by HVPE with nitrogen and hydrogen as carrier gases in the horizontal quartz reactor. HCl diluted by nitrogen reacted with liquid Ga at 850 °C to form GaCl gas, which was then transported into the growth zone where it directly reacted with NH_3_ at 930 °C for 3 min growth of buffer layer to acquire more voids. Here, the initial growth started from the gaps of CNTs, which acted as the nano-mask of selective growth and blocked dislocation propagation. Then, the temperature was maintained at 1050 °C for the subsequent growth of thick films with a growth rate of approximately 80 μm/h. In the growth procedures, the NH_3_ flow rate was held at 1 L/min, and the HCl flow rate was held in the range 30–60 ml/min. The thick GaN film was cooled down to the room temperature, and the self-separation of the GaN layer from sapphire occurred during cooling-down, following the weak connection of GaN and sapphire near the interface.

Scanning electron microscopy (SEM) (JSM-4800LV), Nanoscope III atomic force microscopy (AFM), transmission electron microscopy (TEM), cathodoluminescence (CL) and optical microscopy were used to analyze the structural properties and emission characteristics of the films by plane view and cross-sectional observations. Hall effect measurements were performed at room temperature using the van der Pauw geometry. The crystalline quality of GaN film was evaluated using high resolution x-ray diffraction (XRD) (Bede D1). Photoluminescence (PL) of GaN film was measured at 10 K to address the optical properties and residual strain with the 325 nm He-Cd laser. The stress distribution was also measured using a JY-HR8000 Raman spectrometer with 532 nm excitation.

## Results and Discussion

**Figure 2:**
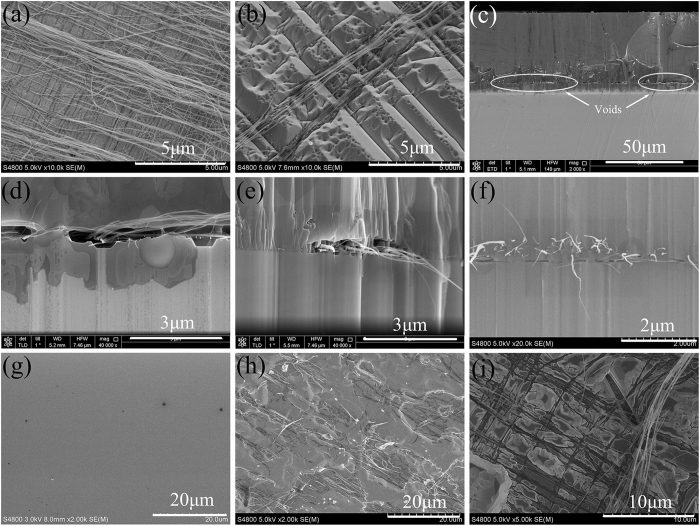
(**a**) Plan-view SEM image of surface morphology of CSCNTs at 930 °C under NH_3_ environment. (**b**) GaN buffer layer on CSCNTs sapphire. (**c**) Cross sectional image of 40-μm-thick GaN film on CSCNTs sapphire using the N_2_ as carrier gas and (**d**) its magnification near the boundary of CNTs and GaN. (**e**) The corresponding boundary of CNTs and GaN on single layer CNTs sapphire. (**f**) The boundary of CNTs and GaN on CSCNTs using H_2_ carrier gas. (**g**) The top surface and (**h**) back-side surface of FS-GaN substrate. (**i**) The left sapphire surface after the self-separation of GaN substrate.

Figure 2(a) shows the surface morphology of CSCNTs at 930 °C under NH_3_ treatment. There are no obvious changes for CSCNTs after nitridation. Subsequently, an ~2-μm-thick intermediate temperature buffer layer is grown on CSCNTs coated sapphire, as shown in Fig. 2(b). It is noted that many grooves and GaN islands have been formed on the CSCNTs template, determined by the intervals between CSCNTs bundles. The CNTs layer has high chemical inertia and doesn’t react with reactant gases, and CNTs thus act as nanomasks for selective area epitaxial growth of GaN in the gaps between CSCNTs bundles. Following the fast growth of HVPE-GaN at 1050 °C, these grooves and islands rapidly coalesce and flat surfaces are formed. During the coalescence, lots of voids are produced at the interface, encircling the CSCNTs bundles, which can be shown after half an hour growth of GaN thick films as seen in Fig. 2(c,d). Clearly, many large voids appear at the boundary of GaN/CSCNTs/GaN in films with a thickness of 40 μm grown in N_2_ carrier gas, while there are small voids on the single layer CNTs template due to the absence of the gridding structures, see the inset of Fig. 2(e). On the other hand, it fails to form large voids at the interface using H2 carrier gas, even on the CSCNTs in Fig. 2(f). The refilled voids are attributed to the low lateral growth rate when using H_2_ carrier gas^23^, which results in the sufficient coalescence along the growth direction. Nevertheless, there is no separation for 40-μm-thickness films no matter which carrier gas is used. When the grown GaN film exceeds a thickness of about 200 μm using N_2_ carrier, FS GaN substrates may be obtained by self-separation after decreasing the temperature, which is possible due to lots of voids and bowing. Despite the irregular spaces between CSCNTs, the separation of uniform FS-GaN substrate can be realized due to enough voids formed near the interface. Figure 2(g,h) show the top and back-side surface morphology of FS-GaN substrate, respectively. The top surface is very smooth and no micro-cracks are observed. In the AFM images, a stepped/terrace structure can be seen on the film surface, indicating step-flow growth mode in spite of high growth rate. In the 10 × 10 μm^2^ areas, the root-mean-square (RMS) roughness of the FS-GaN substrate is about 1.1 nm. On the contrary, the back-side surface of FS-GaN substrate is rough and there are some residual CNTs, which are almost only aligned along the same direction. However, we can also see the grid morphology of GaN on the left sapphire surface after the detaching of GaN-substrate and the CSCNTs still exist as shown in Fig. 2(i). The partial CNTs with one direction on the back-side surface of FS-GaN provide evidence of self-separation mostly occurring near the interface of the upper lay CNTs, followed by the large voids structures. Thermal stress-induced cracks are initiated at the CSCNTs interface and propagate all over the films, producing the total separation of FS-GaN.

**Figure 3:**
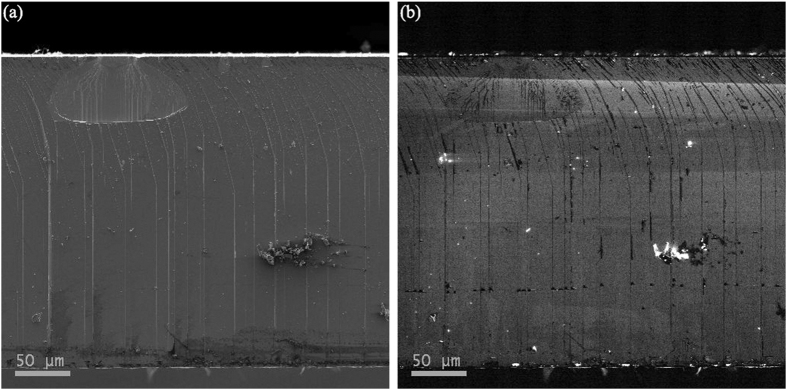
(**a**) The cross-section SEM image of the FS-GaN substrate and (**b**) panchromatic CL image of the same region.

Figure 3(a,b) show the cross-section SEM and corresponding panchromatic CL images of the separated FS-GaN substrate. According to the CL image, the cross-section of FS-GaN with a thickness of 280 μm, demonstrates uniform emission characteristics. It does not reveal any highly radiative columns, even near the back-side surface, which implies CNTs don’t produce a high doping region in the GaN film^24^. The uniform CL emission image represents to the good electric properties of FS-GaN substrate. According to the Hall measurement, the carrier density and mobility of the FS-GaN substrate are 1.2 × 10^18^ cm^−3^ and 216 cm^2^ · V^−1^ · s^−1^, respectively. The background electron concentration of FS-GaN is mainly caused by oxygen and carbon impurities, introduced in the HVPE epitaxial growth^25^,^26^.

**Figure 4:**
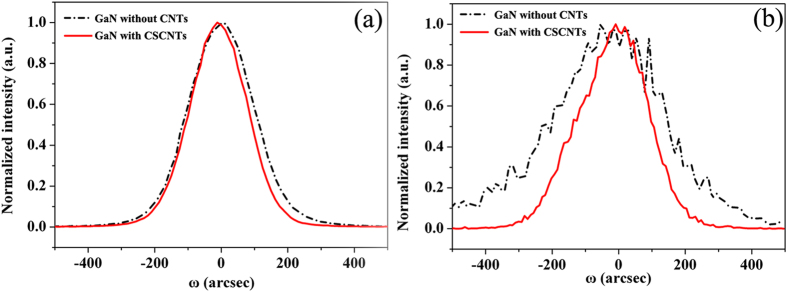
(**a**) (002) and (102) plane rocking curves of 40-μm-thick GaN films without CNTs and with CSCNTs.

Figure 4(a,b) show the HRXRD rocking curves for the 40-μm-thick GaN films without CNTs and with CSCNTs, respectively. The ω-scan gives full widths at half maximum (FWHM) of 230 arcsec (002 reflection) and 352 arcsec (102 reflection) for the GaN film without CNTs. The FWHMs for the GaN film with CSCNTs indicate an obvious decrease for the (002) and (102) reflections, only 200 arcsec and 243 arcsec, respectively. It has been reported that the density of screw dislocations including the screw component of mixed dislocations and edge dislocations including edge components of mixed dislocations correspond to the DCXRD full widths at half maximum (FWHM) of (002) and (102) planes, respectively^27^. The densities of screw-type dislocations D_s_ and edge-type dislocations D_e_ can be estimated from the XRD FWHM values using the following formulas:^28^


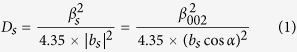



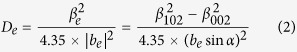


where |b_s_| and |b_e_| are the Burgers vector magnitudes of the screw-type dislocations (|b_s_| = 0.5185 nm) and edge-type dislocations (|b_e_| = 0.3189 nm)^29^, respectively, and β_002_ and β_102_ are XRD FWHM values for (002) and (102) planes, respectively. α is the angle between the reciprocal lattice vector (K_hkl_) and the (001) surface normal. The calculated total dislocation density is 1.2 × 10^9^ cm^−2^ for GaN film without CNTs, while it is only 5.1 × 10^8^ cm^−2^ for GaN with CSCNTs. It is worth noting that edge-type dislocations can be effectively reduced by nano-scale lateral epitaxial overgrowth (NLEO) between CSCNTs. Furthermore, for the self-separated FS GaN film with a thickness of 280 μm, the FWHMs are 162 arcsec and 179 arcsec for (002) and (102) reflections, respectively. It reveals a dislocation density of 2.6 × 10^8^ cm^−2^ for the FS-GaN substrate, further demonstrating the better crystalline quality.

**Figure 5:**
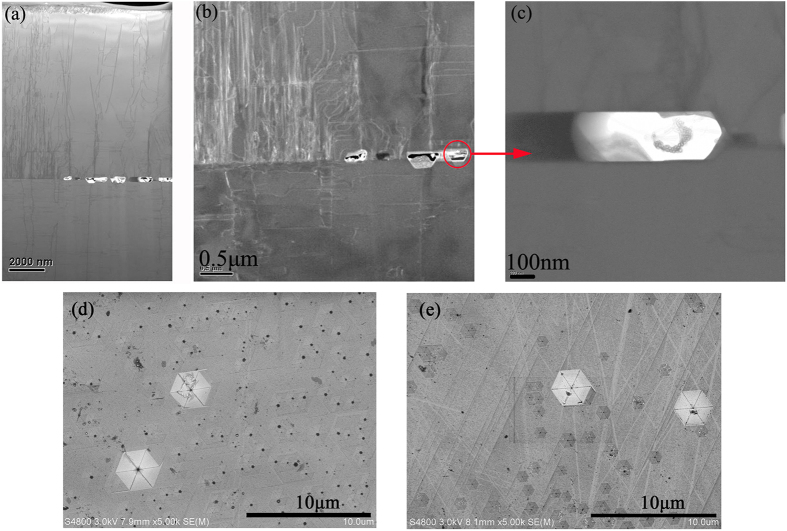
(**a**) Cross-sectional TEM image of HVPE-grown GaN film with CSCNTs. (**b**) Dark field image of GaN near CNTs along the [11–20] zone axis with g vector parallel to the [01–10] direction. (**c**) Magnified TEM image around GaN/CNTs/GaN boundary. SEM images of surface morphology of 40-μm-thick GaN films (**d**) without CNTs and (**e**) with CSCNTs after etching in boiled KOH for 2 min.

In order to understand the mechanism of improved crystalline quality in more details, the transmission electron microscopy (TEM) was employed. Figure 5(a) shows the cross-sectional TEM image of a GaN thick film grown on the GaN template with CSCNTs. It is found that overgrowth of GaN occurs on the area between CSCNTs, and it rapidly coalescences to form a continuous layer. One can observe that some new threading dislocations (TDs) are produced during HVPE growth, especially in the window region between the CSCNTs, whereas many voids and corresponding dislocation annihilation processes are clearly revealed around the CNTs. Figure 5(b) shows a dark field TEM image of GaN along the [11–20] zone axis with g vector parallel to the [01–10] direction. Edge and mix type dislocations are visible under this diffraction geometry. Obviously, many dislocations with an edge component are bent around and annihilate at the climax of voids. Here, similar to the previous SiO_2_ nanorod arrays embedded in GaN epilayers^30^,^31^, the CSCNTs may act as nano-masks to prevent the TDs from going through to the upper epilayer. At the initial growth stage, the CSCNTs impact the distribution of the GaN islands and subsequently GaN islands are coalesced directly in the lateral growth mode. The TDs arising at the coalescence boundaries^32^ may be bent over due to the native masks of CNTs. In addition, air voids formed around CNTs also have a similar blocking effect. Thus, they suppress the propagation of TDs during the lateral coalescence, resulting in a high quality GaN epilayer. Meanwhile, CNTs are stable at high temperatures and no reactors adhered onto the CNTs as shown in Fig. 5(c). Thus, no other crystal grains and coalescence boundaries appear around the CNTs, which guarantees the high quality single-crystal GaN. Furthermore, we also adopt boiled KOH as the etchant to evaluate the dislocation density of the GaN films. As shown in Fig. 5(d,e), surface etch-pit density is about 2.6 × 10^7^ cm^−2^ for 40-μm-thick GaN film without CNTs, with respect to 1.2 × 10^7^ cm^−2^ for 40-μm-thick GaN with CSCNTs. Weyher *et al*. had presented that in the etched GaN in KOH, the larger pits are mainly formed on screw, and the smaller pits are on the edge-type dislocations^33^. Obviouly, CNTs are beneficial to the reduction of dislocations during NLEO, especially edge-type dislocations. This illustrates the consistent trend with the previous XRD results.

**Figure 6:**
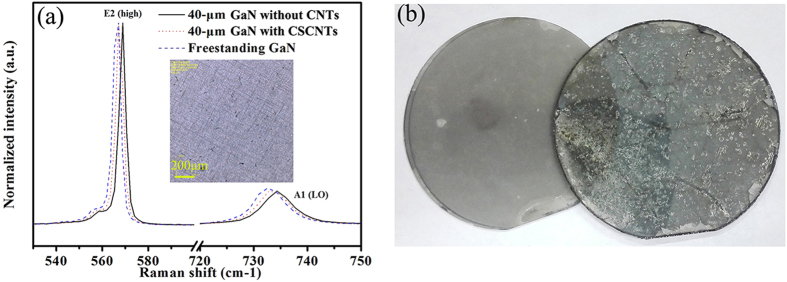
(**a**) Raman spectra of 40-μm-thick GaN films without CNTs and with CSCNTs and FS GaN substrate. The inset shows the optical microphotograph of 40-μm-thick GaN film with CSCNTs. (**b**) Photograph of FS-GaN substrate self-separated from sapphire by CSCNTs.

Besides improved crystalline quality, CNTs also help to release the compressive stress in the GaN films and thus produce the win-win situation. Figure 6(a) shows the room-temperature Raman spectra of 40-μm-thick GaN films without CNTs and with CSCNTs and FS GaN substrate after self-separation. The E_2_-high phonon modes obtained from 40-μm-thick GaN films without CNTs and with CSCNTs are located at 568.8 and 567.5 cm^−1^, respectively. In comparison, the E_2_ phonon frequency of FS-GaN is 566.4 cm^−1^, which is very close to that of the stress-free bulk GaN substrate, which is believed to be 566.2 cm^−1 ^34. The E_2_ phonon frequency is sensitive to the strain and has been extensively used in the characterization of GaN to quantify stress using the following equation:^35^


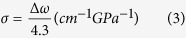


where σ is the residual stress and Δω is the E_2_ phonon peak shift. Thus, it is calculated that thick films obtain a stress relaxation of 0.31 GPa by CSCNTs and there is nearly no stress in the separated FS-GaN substrate. As shown in the inset of Fig. 6(a), CSCNTs may clearly be observed and some trenches also occur due to the fast coalescence. The absence of cracks in the 40-μm-thick GaN film implies that the voids effectively help to release the tensile stress due to thermal mismatch. On one hand, such CSCNTs layers should weaken the interface. On the other hand, most of the stress during cooling-down should arise exactly at this position, otherwise the cracking may occur at an undefined position in the crystal. Finally, 2-inch self-separated FS-GaN substrates are achieved and Fig. 6(b) presents the separated surface of FS-GaN and sapphire. When the critical thickness of the GaN film is reached, interface cracking ensues between the CSCNTs and voids structure during cooling down due to the thermal stress, leading to the separation of the GaN from the sapphire substrate, which is also observed in Fig. 2(h,i). Lots of voids and weak connection between CSCNTs guarantee the complete separation. However, there are still some cracks observed at the interior of FS-GaN substrates and further growth optimization needs to be carried out to realize the crack-free GaN substrates.

**Figure 7:**
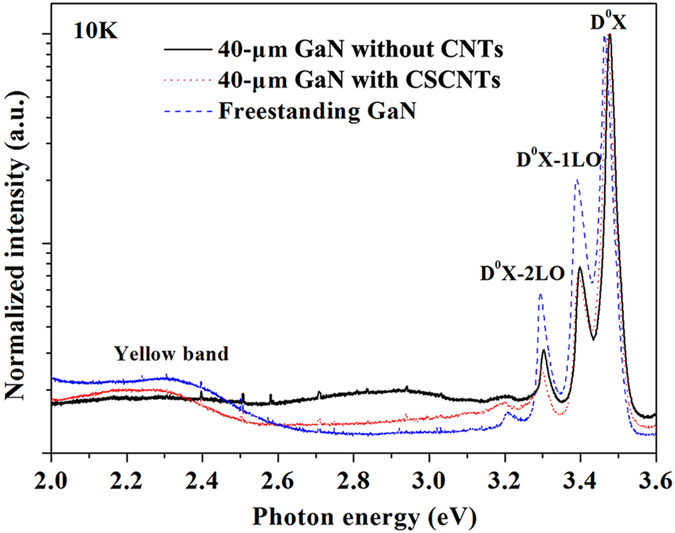
PL spectra of 40-μm-thick GaN films without CNTs and with CSCNTs and FS-GaN substrate at 10 K.

To assess the optical properties of these GaN films, low temperature PL spectra of GaN films and FS-GaN substrates are also measured at 10 K, as shown in Fig. 7. All the PL spectra are dominated by donor bound exciton (D^0^X) emission and its longitudinal-optical (1LO and 2LO) phonon replicas. Compared to GaN film without CNTs, the D^0^X peak is redshift by 5 meV for the GaN film with CNCNTs and by 13 meV for FS-GaN substrate, respectively, revealing the relief of the compressive stress in the GaN. Furthermore, the FWHM of D^0^X peak obtained from the multi-Gaussian fitting is about 8.1 meV for the FS-GaN substrate, which is narrower than that from the GaN film without CNTs (14.9 meV), demonstrating improvement in crystalline quality. In addition, for the FS-GaN substrate, it looks black due to the existence of CNTs as shown in Fig. 6(b). However, CNTs don’t deteriorate the optical property of GaN substrate. Usually, the yellow luminescence is known to be related to the impurities or the defect^25^,^36^. Here, the yellow band of FS-GaN substrate is slightly increased compared to that without CNTs, keeping the high intensity ratio of D^0^X/yellow band.

The CNTs layer is critical for the fabrication of the self-separated GaN substrates. Although such nanomaterial is new for GaN epitaxy, the mass production of the CNTs materials with low-cost has been realized on 8-inch Si wafer and these CNTs have been successfully applied in the commercialized CNTs touch panels. The controllable costs can be attributed to both the raw materials and the film processes. Acetylene is the main raw material used for the CNT synthesis, which is abundant, cheap and widely used in industry. Furthermore, the spinning and coating procedure are also efficient, as we have achieved the automation of these procedures. In addition, the dry spinning process is environmentally friendly. Thus it can be expected that patterning substrates with CNTs will be an efficient and low-cost technique for the high-quality GaN epitaxial growth^37^.

## Conclusions

Self-separated 2-inch GaN substrates are successfully realized using CSCNTs coated sapphire by HVPE growth. The x-ray diffraction, TEM and wet-etching results show nano-scale selective epitaxial growth on CSCNTs obviously improve the crystalline quality of GaN film. Furthermore, it effectively helps to release the stress as a result of the void formation. The weak interface connection between GaN films and sapphire substrate due to the occurrence of air voids guarantees the GaN film self-separation during cooling-down. In addition, CNTs don’t deteriorate the optical properties of GaN and the FWHM of D^0^X peak is 8.1 meV for self-separated FS-GaN substrate, demonstrating improved crystalline quality.

## Additional Information

**How to cite this article**: Wei, T. *et al*. Cross-stacked carbon nanotubes assisted self-separation of free-standing GaN substrates by hydride vapor phase epitaxy. *Sci. Rep.* **6**, 28620; doi: 10.1038/srep28620 (2016).
